# Problems with the mapping of magic tricks

**DOI:** 10.3389/fpsyg.2015.00855

**Published:** 2015-06-23

**Authors:** Peter Lamont

**Affiliations:** School of Philosophy, Psychology and Language Sciences, University of EdinburghEdinburgh, UK

**Keywords:** science of magic, magic, theory, wonder, methodology

A few years ago, colleagues and I (Lamont et al., 2010) argued that a “science of magic” was misguided. As we said then, the problem is not with using conjuring knowledge to explore psychological processes. This has been done for well over a century, and is of obvious potential value. The problem is with the grander aim of constructing a general scientific theory of magic (p. 20). It is this kind of “science of magic” that was being proposed then, and it is still being proposed now. Rensink and Kuhn (2015) seek “natural” inventories and taxonomies of magic tricks, which would serve as a basis for a scientific theory of magic, and which could be used to describe the relationships between effects and methods (pp. 8–9). The purpose of this brief paper is to explain why, in my opinion, this aspect of their approach remains problematic.

Rensink and Kuhn (2015) argue that magic tricks should be treated as objects of scientific investigation in their own right, which might be studied in terms of their specific components, and which might be explained in psychological, neural and functional terms. They also argue that magic tricks could be studied in terms of the relationships between effects and methods. To do this, they argue, a complete inventory of magic tricks is needed. In order to maintain the many-to-many relationships between effects and methods, they suggest that separate effect-centered and method-centered inventories could be constructed, and that more natural taxonomies (like those that emerged in chemistry) might follow (pp. 14–23).

In principle, this may sound plausible enough, but how might it proceed in practice? Certainly, a list of magic tricks can be constructed, and a variety of relationships between effects and methods might be described. After all, this has been done many times already (for some examples, see Lamont and Wiseman, 1999, pp. 1–7). But how might one construct a complete inventory, or a more natural taxonomy, of magic tricks? How might such an approach provide an understanding of the relationships between effects and methods?

In order to illustrate certain problems, I will begin with the authors' own exemplar of a trick with an effect and a method, the so-called “Materializing Card” (p. 6). In this trick, according to the authors, a deck of cards is riffled toward a spectator, who is asked to name a card; the magician then produces this card from a pocket. Now, what type of effect is this? It could be presented as a “transposition”: a selected card is transported from the deck to the magician's pocket. It could also be presented as a “prediction”: someone names a card, and a matching “prediction” is removed from the magician's pocket. The “Materializing Card” might be either, depending on how it is performed, and a handful of words could make the difference. For example, by saying: “… and now your card is in my pocket,” then removing the card from the pocket, the trick becomes a transposition. Alternatively, by saying: “… I predicted that you would choose that card,” then removing the card from the pocket, the trick becomes a prediction.

This is one problem: effects do not naturally slot into categories, because many can be presented as more than one kind of effect. Indeed, even at their most abstract level, many types of effects overlap. For example, most transpositions could be presented as two effects—a vanish (from one place) and an appearance (elsewhere)—and most predictions could be presented as clairvoyance, mind-reading or mind control (Lamont and Wiseman, 1999, pp. 7–27). In other words, placing magic tricks into mutually exclusive categories is fundamentally problematic.

Now, let us consider the relationship between effect and method: what is the method of the “Materializing Card”? According to the authors, it is a specific kind of riffle force that relies on the timing of the riffle. The authors identify this timing as a “key factor” (p. 6) in the method of this trick. However, a similar effect can be achieved by countless other methods. There are other kinds of riffle force, which depend on the timing of the riffle, but not in the same way as the authors' chosen method. For example, the riffle might be timed to coincide with when the spectator says “stop,” but this kind of timing is difficult to isolate from live human interaction. There are also countless further ways to force a card that involve neither timing nor riffling the cards. Furthermore, a similar effect could be achieved without forcing the card at all. For example, the spectator could freely choose a card, and a matching card could be removed from a pocket by using a card index. Alternatively, the freely selected card could be stolen from the deck, palmed and then apparently removed from a pocket.

In other words, there are countless ways to achieve a similar effect in which the key factor identified by the authors—the timing of the riffle of the cards—is simply irrelevant. It is only “key” to the specific type of force chosen by the authors. This particular force has been chosen because it is ideal for experimental study. The authors have identified a particular technique that is amenable to experimental enquiry, because it can be tested by showing a video to participants, and manipulating the timing of the riffle. That is fair enough. However, a comprehensive list of methods for this single effect would have to include, among other things, all methods of forcing a card, and all methods of surreptitiously stealing a card from the deck. Every one of these methods depends, in turn, on various other specific factors. All of these methods could also be used in (literally) countless other effects. Not to mention that new methods are continually being invented. In other words, there are an endless number of relationships between effects and methods.

So, let us attempt something much simpler: a comprehensive list of effects, regardless of presentation or method. What might this look like? Let us start, again, with the “Materializing Card.” Now, imagine precisely the same effect, but when the magician removes the card from the pocket, it is inside an envelope. This is consistent with the authors' description, but is it the same effect? In conjuring terminology, is this “card to pocket” or “card to envelope”? If the envelope were on the table from the start of the trick, this would clearly be “card to envelope,” but is this the same effect as the other “card to envelope”? What if the card was inside a wallet (perhaps inside an envelope, which was inside the wallet), which might be in a pocket, or on the table, or in the hands of the spectator: how many different effects would this be? That, of course, is a highly subjective matter, as would be any decision to distinguish between effects according to particular details. For example, from which pocket is the card removed: the magician's trouser pocket that is concealed by the jacket, or an outside jacket pocket that is in full view throughout the trick, or the spectator's own pocket? Does it matter if the card is signed by the spectator at the start of the trick (in order to show, later, that a duplicate card is not being used)? At what point, and on what basis, do we decide that one effect becomes another?

Meanwhile, a chosen card might reappear in some other location, such as inside the card box, or some other kind of box, or inside a balloon, a cigarette, a walnut, a lemon, an orange, a tin of peaches … I am not making this up, all these tricks have actually been performed. Think of a location, any location, and chances are that a magician has, at some point, made a selected card appear there. The possibilities are never-ending, as would be any list of effects involving a card vanishing and reappearing somewhere else. To this, of course, must be added all other card effects. And then all other tricks with all other objects. There are also countless mentalist effects that do not involve any objects. Not to mention that new effects are always being invented. Meanwhile, what about the old tricks? How many “cups and balls” should be on the list, since they use different numbers of cups and balls, of different shapes and sizes, and have different phases, and different numbers of phases, and are performed in different contexts in different ways?

The problem is not simply that the list would be interminable. The problem is more fundamental: since any effect can be performed in many ways, at what point does it become a different effect? Since there are no clear boundaries, we need to make a host of decisions about how to construct any list of magic effects. We cannot possibly list every variation, so we need to decide which differences matter, and which do not. Is “card to lemon” not essentially the same effect as “card to orange”? But is it essentially the same as “bill to lemon,” in which a bill (banknote) disappears and reappears inside a lemon? Bills also reappear inside oranges and envelopes because, in magic, they often serve much the same function as a playing card. They also reappear inside bananas. However, at what point on the slippery slope from “card to lemon” to “bill to banana” do we draw a line? And, just as importantly, *where* do we draw the line: between cards and bills, or between lemons and bananas? In other words, do we categorize on the basis of object (cards, bills, etc.) or location (lemons, bananas, etc.)? As it happens, many “cups and balls” routines also end with the production of fruit: lemons, oranges, sometimes a melon, and I have seen the occasional tomato (which is also a fruit, scientifically speaking, based on a natural set of criteria).

This is the fundamental problem of constructing a list of magic tricks. We have to make decisions about what constitutes a distinct trick, and what constitutes a different version of the same trick. We have to do this on the basis of particular criteria. There is no obvious reason for choosing one set of criteria rather than another. However, depending on the choice we make, this will result in one list rather than another. Since there are so many choices to be made, we end up with so many different lists. That is precisely why we have divided magic tricks into so many different lists since the sixteenth century. They have been categorized according to effect and method, props and presentational style, audience and venue. Even when they have been divided into general kinds of effects, there have been many different lists, because even this is a remarkably subjective matter (Lamont and Wiseman, 1999, p. 2).

The recent taxonomy of misdirection (Kuhn et al., 2014), which the authors “hope might provide a template for other aspects of magic more generally” (p. 13), does not solve the problem. In this paper, the authors divide misdirection into three psychological categories: perception, memory and reasoning. This is certainly another way of understanding misdirection, but it is far from clear why this taxonomy is “more natural” (p. 1). It is merely another way of arranging remarkably fuzzy concepts, in this case by making an initial division into perception, memory and reasoning. This particular division makes sense from a psychological perspective, but it merely begs the question: why not simply use magic to investigate perception, memory and reasoning? After all, that is what psychologists have done successfully so far, and we do not need a taxonomy of misdirection, or of magic tricks, to do this.

In conclusion, it is certainly possible to construct another inventory of magic tricks, and to describe a variety of relationships between effects and methods. However, it is difficult to see how a complete list of magic tricks could be compiled, or why any list might be “more natural” than another. If compiling a list of magic tricks is problematic, identifying relationships between effects and methods would be an endless process, since a single trick, such as the authors' own exemplar, can be categorized as different effects, and can be performed using hundreds of different methods. Meanwhile, the value of such efforts is unclear, since we do not need such a framework to study psychological processes. We can continue to use magic to study psychological processes in a variety of ways, including some of the other ways that the authors have suggested. This, in my opinion, is a more useful direction to take.

## Conflict of interest statement

The author declares that the research was conducted in the absence of any commercial or financial relationships that could be construed as a potential conflict of interest.
